# Sex Chromosome Equality in Plants

**DOI:** 10.1371/journal.pbio.1001312

**Published:** 2012-04-17

**Authors:** Robin Meadows

**Affiliations:** Freelance Science Writer, Fairfield, California, United States of America

## Abstract

Evidence for dosage compensation in *Silene latifolia*,a plant with 10-million-year-old sex chromosomes, reveals that dosage compensation can evolve rapidly in young XY systems and is not an animal-specific phenomenon.

**Figure pbio-1001312-g001:**
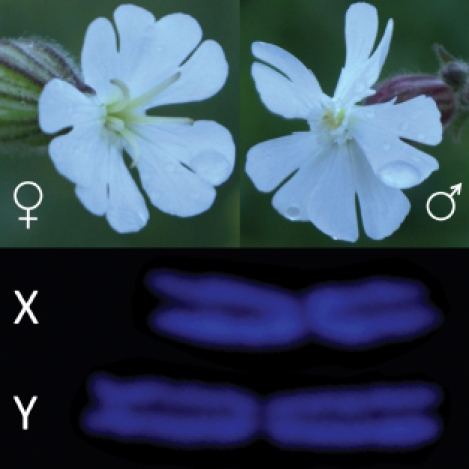
Pictures of female and male flowers (top) and the X and Y chromosomes (bottom) in *Silene latifolia*. Image credit: Jos Käfer and Roman Hobza.

[Fig pbio-1001312-g001]Birds do it, bees do it—and now it appears that even plants do it. No, it's not what you're thinking. Sexual reproduction is obviously well-known in plants. Rather, the phenomenon in question is equalizing sex chromosome expression in males and females. In animals where males are XY and females are XX, the Y chromosome has lost most of its genes, so X chromosome expression is adjusted to keep the balance between sexes. In people, females inactivate one of their X chromosomes. The fruit fly *Drosophila melanogaster* takes the opposite approach, with males hyper-expressing their X-linked genes. Called dosage compensation, such balancing is found in many animals but was thought to be absent in plants. Now, however, in this issue of *PLoS Biology*, Gabriel Marais and colleagues report the first evidence of dosage compensation in the plant *Silene latifolia*, or white campion, along with insights into how this phenomenon evolves.

As in mammals and fruit flies, *S. latifolia* males are XY and females are XX. The classical view holds that there are three steps in the evolution of dosage compensation: recombination between the X and Y chromosomes is suppressed, the Y degenerates, and this massive loss of Y chromosome genes is balanced by dosage compensation of the X chromosome. But animal sex chromosomes began evolving so long ago that the process is too advanced to trace its course. Human sex chromosomes originated about 150 million years ago, for example, and the Y chromosome has now lost some 97% of its genes. In contrast, sex chromosomes are still in the early stages of evolution in *S. latifolia*, making it a good system for studying this process.

Like animal sex chromosomes, *S. latifolia* X and Y chromosomes have gradually stopped recombining and the Y chromosome is degenerating. However, work on sex chromosome evolution in this plant has been limited by the small number of known sex-linked genes. To identify more *S. latifolia* sex-linked genes, the researchers used a new technique called RNA sequencing, which both sequences and estimates the abundance of mRNAs. They identified more than 1,700 sex-linked genes, a 100-fold increase on the number previously known. Next, the researchers assessed degeneration of the *S. latifolia* Y chromosome by comparing expression levels of X- and Y-linked genes in males. In keeping with research on the previously known sex-linked genes, the results confirmed that the average expression levels of Y-linked genes were lower than those of their X-linked counterparts.

Reduced expression fits with the on-going degeneration of the Y chromosome, raising the question of whether *S. latifolia* also has dosage compensation. The researchers tested this by comparing the expression of sex chromosome-linked genes in males and females. If *S. latifolia* lacked dosage compensation, the expression of X-linked genes in males would be half that seen in females. Indeed, this is true for genes where the Y-linked copy is still expressed. However, for genes with reduced Y expression, expression of the X-linked copy was nearly as high in males as in females. This suggests that, like fruit flies, *S. latifolia* compensates for reduced Y expression by increasing X expression in males. The researchers then excluded sex chromosome genes that are expressed more in males than in females. Such male-biased genes are not subject to dosage compensation in other species, and comprised 25% of the newly identified sex-linked genes in *S. latifolia*. Analysis of expression levels of the remaining 75% revealed that in males, expression of the X-linked version of a gene rises in proportion to the drop in expression of the Y-linked version, bolstering the conclusion that dosage compensation is gradually evolving in *S. latifolia* in piecemeal fashion.

The finding that *S. latifolia* balances X chromosome expression between the sexes contradicts a recent study by another research team (Chibalina and Filatov [2011] Curr Biol 21: 1475), which concluded that *S. latifolia* lacks dosage compensation. But Marais and colleagues argue that, if analyzed differently, the results of the previous study may actually support dosage compensation as well. The other team based their conclusion on the fact that X expression levels were not equal in males and females. However, Marais and colleagues point out that the X expression level in males was still considerably higher than would be expected in the absence of dosage compensation. Instead of being half as high, the average X expression level in males was nearly 70% that of females, suggesting that there is dosage compensation for many genes. Moreover, partial dosage compensation is to be expected in a system where it is still evolving.

Besides being the first to support the existence of dosage compensation in plants, this work provides the evolutionarily earliest example of balanced X expression between the sexes. In animals, dosage compensation has been found only in sex chromosome systems that are more than 100 million years old, while *S. latifolia*'s sex chromosomes are just 10 million years old. The discovery of dosage compensation in such young sex chromosomes affords a unique and exciting opportunity to learn how this phenomenon might evolve.

**Muyle A, Zemp N, Deschamps C, Mousset S, Widmer A, et al. (2012) Rapid De Novo Evolution of X Chromosome Dosage Compensation in** ***Silene latifolia*** **, a Plant with Young Sex Chromosomes. doi:10.1371/journal.pbio.1001308**

